# Predictive Modelling and Optimisation of Rubber Blend Mixing Using a General Regression Neural Network

**DOI:** 10.3390/polym17131868

**Published:** 2025-07-03

**Authors:** Ivan Kopal, Ivan Labaj, Juliána Vršková, Marta Harničárová, Jan Valíček, Alžbeta Bakošová, Hakan Tozan, Ashish Khanna

**Affiliations:** 1 Department of Numerical Methods and Computational Modelling, Faculty of Industrial Technologies in Púchov, Alexander Dubček University of Trenčín, Ivana Krasku 491/30, 020 01 Púchov, Slovakia; ivan.kopal@tnuni.sk (I.K.); ivan.labaj@tnuni.sk (I.L.); juliana.vrskova@tnuni.sk (J.V.); alzbeta.bakosova@tnuni.sk (A.B.); 2 Department of Electrical Engineering, Automation and Informatics, Faculty of Engineering, Slovak University of Agriculture in Nitra, Tr. A. Hlinku 2, 949 76 Nitra, Slovakia; jan.valicek@uniag.sk; 3 Department of Mechanical Engineering, Faculty of Technology, Institute of Technology and Business in České Budějovice, Okružní 10, 370 01 České Budějovice, Czech Republic; 4 Department of Industrial Engineering, College of Engineering and Technology, American University of the Middle East, Egaila 54200, Kuwait; hakan.tozan@aum.edu.kw; 5 Department of Computer Science and Engineering, Maharaja Agrasen Institute of Technology, GGSIP University, New Delhi 110078, India; ashishkhanna@mait.ac.in

**Keywords:** elastomers, rubber blends, mixing process, intelligent modelling, general regression neural network, optimisation

## Abstract

This paper presents an intelligent predictive system designed to support real-time decision making in the control of rubber blend mixing processes. The core of the system is a General Regression Neural Network (GRNN), which accurately predicts key process parameters, such as viscosity (expressed as torque), temperature, and energy consumption across varying masses of the processed material. The model can evaluate the mixing progress based on the initial 10% of input data, allowing early intervention and process optimisation. Experimental validation was conducted using a Brabender Plastograph EC Plus with a natural rubber-based blend in the mass range of 60–75 g. The GRNN kernel width parameter (σ) was optimised through a 10-fold cross-validation. High predictive accuracy was confirmed by values of the coefficient of determination (R^2^) approaching 1, and consistently low values of the root mean square error (RMSE). This system offers a robust and scalable solution for intelligent process control, productivity enhancement, and quality assurance across diverse industrial applications, beyond rubber blending.

## 1. Introduction

Mixing elastomeric blends is a critical step in rubber manufacturing that directly influences the properties of the final product [1]. This complex process, which requires the uniform dispersion of ingredients throughout the blend volume, is challenged by the intrinsic properties of the materials involved. Elastomer melts behave as non-Newtonian fluids with viscosity dependent on shear conditions, whereas reinforcing fillers exhibit surface activity characteristics of solids, significantly hindering their dispersion within the elastomer matrix [2]. During mixing, friction-generated heat increases the blend temperature, reduces the elastomer viscosity, and promotes filler distribution [3]. However, an excessively low viscosity can cause filler agglomeration and phase segregation, leading to defects in the final product. Conversely, overly high temperatures can cause premature vulcanization or thermal degradation of blend components [4]. The mixing outcome depends not only on the properties, amounts, and proportions of the components but also on numerous other factors [5].

A major challenge in the rubber industry is balancing cost reduction and productivity gains while maintaining blend quality. Cost efficiency is often pursued via greener production methods, whereas productivity improvements involve increasing the batch mass per mixing cycle within the optimal fill factor limits of mixing equipment [6]. Processing larger material quantities in the same time frame increases the molecular friction, potentially raising the temperature and lowering the blend viscosity, adversely affecting the rheological and thermal properties. Thus, continuous monitoring of the temperature, viscosity, energy consumption, and mixing time of various blend masses is essential. While industrial mixers typically offer such monitoring, they lack reliable analytical models to ensure proper process progression, as many models fail to capture the dynamic variability influencing the mixing parameters and final product quality [7,8,9].

Consequently, increasing rubber production productivity without significant equipment increases the risks of compromising blend quality. Artificial intelligence (AI), particularly machine-learning (ML)-based artificial neural networks (ANNs), offers promising solutions by enabling real-time analysis of process parameter effects using historical data [10]. ANNs excel at mapping complex relationships between variables and applying learned patterns to new data, making them invaluable for the predictive modelling, control, and optimisation of dynamic manufacturing processes [11,12,13,14,15,16,17,18].

For rubber blend mixing, well-trained ANNs integrated with mixer hardware can form flexible closed-loop systems for the precise regulation and optimisation of the process [19]. However, ANN-supported intelligent rubber mixing remains underexplored in current research. Recent studies have addressed various aspects, and an analytical overview of recent applications of machine learning models in materials engineering was provided in ref. [20]. A previous study [21] compared effective strategies for implementing ANNs in the predictive modelling of the rheological behaviour of materials, including rubber blends. The system architecture of an ANN for the automated identification of temperature deviations during rubber blend mixing, based on formulation and batch mass, is presented in ref. [22]. A novel ANN model for generating the so-called temperature trajectory inside a mixer, aimed at assessing the quality of the mixing process, was introduced in ref. [23]. A study [7] proposed an online deep ANN-based model for predicting rubber blend quality during mixing using the degree of carbon black dispersion as a quality index. ANNs also play an important role in optimizing material formulations for additive manufacturing, as demonstrated in ref. [24], where epoxidized natural rubber enhanced the mechanical strength, chemical resistance, and thermal stability of printed parts, with ANN outperforming conventional statistical methods. In addition to elastomers, ANN and ANFIS models have been successfully applied to optimise processing parameters in thermoplastic manufacturing, such as acrylonitrile butadiene styrene (ABS), including the prediction of mechanical properties based on printing conditions [25]. Although representing a different class of polymers, the results confirm the high accuracy of soft computing techniques and highlight their broader potential for application in the processing of elastomeric systems, including rubber compounds.

The General Regression Neural Network (GRNN) is a particularly suitable ANN variant for analysing and predicting time-dependent mixing parameters [26]. Its probabilistic function approximation architecture delivers robust and fast-converging solutions with minimal training. GRNN’s capability to nonlinearly approximate relationships between time-varying inputs and outputs and to handle noisy or incomplete data effectively makes it ideal for real-world dynamic process prediction [18,27]. In rubber mixing, the GRNN facilitates continuous process monitoring and real-time comparison with expected profiles, enabling early deviation detection and production quality stabilization. Despite these advantages, GRNN applications in rubber blend predictive modelling remain unreported.

The main contribution of this study is the development of a novel, fast, and flexible intelligent decision-support system based on a GRNN for real-time monitoring, modelling, and optimisation of rubber blend mixing processes. The model accurately predicted the rotor torque (viscosity), motor current (energy consumption), and blend temperature as functions of the material mass and mixing time based on experimental data from its early phase. The optimised GRNN supports dynamic adjustment of input/output variables and parameter levels, offering a scalable solution adaptable to diverse industrial applications beyond rubber manufacturing.

## 2. Materials and Methods

### 2.1. Studied Rubber Blend

The formulation of the elastomer blend investigated in this study, a commonly used natural rubber-based blend in the rubber industry, is summarized in Table 1, which also specifies the role of individual components and their manufacturers.

To ensure reproducibility and precise dosing of each ingredient according to the specified formulation, conversion from phr units (parts per hundred rubber) to absolute mass in grams was performed when adjusting the rubber matrix content. The conversion is based on the following relationship:

(1) $$G=\frac{m}{\sum_{i=1}^{n}P_{i}},$$

where *G* is the equivalent mass per phr unit in grams, *m* is the total mass of the blend in grams, *P_i_* is the quantity of each individual component in phr, and *n* is the total number of components. The absolute masses of the individual components *m_i_* in grams were then calculated as follows:

(2) $$m_{i}=G\cdot P_{i}.$$

### 2.2. Rubber Blend Mixing

The rubber blend was prepared using a Brabender Plastograph EC Plus laboratory mixer (Brabender GmbH & Co. KG, Duisburg, Germany) equipped with an 80 cm^3^ mixing chamber. One-step mixing was performed in accordance with ISO 2393 [28] with a fill factor ranging from 0.65 to 0.85. The total mass of the prepared blend was varied from 60 to 75 g in 5 g increments.

Although the experimental technique was conducted under laboratory conditions using a relatively small mixing chamber, the proposed predictive system was designed to be scalable and transferable to industrial settings involving substantially larger blend volumes, provided that sufficiently representative process data are available for model training. Because the GRNN learns from input–output data relationships rather than relying on fixed physical equations, the model architecture remains applicable across different scales, as long as the input variables reflect the relevant technological conditions. This aspect of scalability is discussed further in Section 3.4.

The first step of the mixing process involved mastication of the natural rubber matrix, with masses ranging from 42 to 53 g in variable increments, as calculated using Equations (1) and (2). Mastication was performed at a constant chamber temperature of 90 ± 1 °C, under a pressure ram loaded with a 5 kg weight, and at a rotor blade speed of 50 ± 1 rpm, lasting approximately 3 min. Subsequently, zinc oxide (ZnO) was added as a vulcanization activator, followed by 45 s of mixing. Stearic acid, which served as an additional activator, was then incorporated with 30 s of further mixing. Carbon black was introduced as the reinforcing filler and mixed for 3 min. Finally, sulfur and TBBS were simultaneously added as the vulcanizing agent and accelerator, respectively, and blended for an additional 1.5 min.

The torque and temperature sensors were calibrated prior to each experiment to ensure precise data acquisition. The entire weighing and mixing procedure was repeated five times for each tested batch mass under consistent laboratory conditions—ambient temperature, 23 ± 2 °C; atmospheric pressure, 101.3 ± 2 kPa; and relative humidity, 50 ± 5%. For each of the five repetitions, the torque and blend temperature were recorded over time. Mean values and standard deviations were subsequently calculated. Although no formal statistical experimental design (e.g., Design of Experiments (DOE)) was applied, the number of repetitions was selected to ensure the representativeness and reliability of measurements under controlled conditions. The average standard deviation of torque across the mixing process did not exceed ±5%, and that of the blend temperature remained within ±1.5 °C. These values fall within the acceptable ranges for laboratory-scale experiments, considering the material characteristics and dynamic nature of the mixing process.

Component masses were measured using a Mettler Toledo XS205 precision laboratory scale (Mettler Toledo International Inc., Columbus, OH, USA) with an accuracy of ±0.01 g, ensuring precise dosing of raw materials without introducing significant error into the experimental results.

The mixer captured mixing curves for the rubber blend at various batch sizes, expressed as functional dependencies of the torque and temperature on the mixing time. All measurements were acquired synchronously at a fixed sampling interval of 2 s (0.5 Hz), which provided a sufficient temporal resolution to capture the dynamic evolution of the process. The recorded data were exported in the ASCII format and prepared for use in the GRNN model. In total, 60 time-resolved curves were recorded at two-second intervals—torque, blend temperature, and calculated current—obtained for four batch masses (60, 65, 70, and 75 g), with five repetitions for each. As is common with many industrial mixers, the laboratory mixer does not directly measure power consumption during mixing. To address this technical limitation, the electric current *I* drawn by the motor was estimated from the torque values using the following equations [29]:

(3) $$I=\frac{P}{\sqrt{3}\cdot U\cdot \cos\varphi },$$

where *I* is the electric current (A), *U* is the voltage (V), and *cosφ* is the power factor of the Brabender Plastograph. The input power *P* (kW) is calculated as follows:

(4) $$P=\frac{M\cdot \eta }{9.55},$$

where *M* is the torque (N·m), *η* is the motor speed (rpm), and 9.55 is the conversion factor used to obtain power in kilowatts [30].

The combined analysis of the torque and power consumption has proven to be a reliable indicator of blend homogeneity in rubber processing. Previous research has shown that stabilized torque profiles and decreasing energy consumption over time correlate with improved dispersion and uniformity of components in the blend [19]. Therefore, these process characteristics are appropriate proxies for evaluating the mixing process. Consequently, modelling their evolution using a neural network enables not only the prediction of process behaviour, but also the indirect assessment of blend homogeneity in real time.

### 2.3. Experimental Data Processing

The development of the GRNN model, as well as all data analyses, numerical computations, and visualizations in this study, were performed using MATLAB^®^ R2022a (version 9.12, 64-bit; MathWorks, Natick, MA, USA) with the Neural Network Toolbox and Statistics and Machine Learning Toolbox. The software was installed on a Windows 11 PC (Intel^®^ Core^™^ i5-12450H, 2.4 GHz, 16 GB RAM, NVIDIA GeForce GTX 1650 GPU, 6 GB, 250 GB SSD).

For each mixing cycle, three time-series vectors were recorded: the torque (**M**), blend temperature (**T**), and electric current (**I**), each expressed as a function of the mixing time (**t**). Data pre-processing included the removal of incomplete and erroneous records. Specifically, any instance in which the independent variable lacked a corresponding dependent value was classified as incomplete and was excluded from further analysis. Consequently, clean vectors of **M**, **T**, and **I** were obtained for each blend mass, serving as target outputs for the GRNN model. The model inputs consisted of blend mass **m** (a categorical variable with multiple levels) and mixing time **t**, treated as a continuous independent variable specific to each blend mass. The processed data were structured into cell arrays containing matrix-based tables of input vectors **X** (blend mass and mixing time) and the corresponding target vectors **Y** (torque, blend temperature, and electric current).

All data were normalized to the range ⟨0, 1⟩, ensuring consistent scaling across datasets with different value ranges and magnitudes. This normalization step significantly improves the learning efficiency of the GRNN model, training convergence, predictive performance, and numerical stability [31].

Despite the relatively high level of noise present in all recorded experimental curves, arising from the measurement system, mixing dynamics, and changes in boundary conditions (e.g., repeated opening of the mixer chamber), the time series were not smoothed before the GRNN processing. Among the signal characteristics considered informative, the fluctuation patterns, maximum values, and rising slopes of torque, temperature, and current, while the noise was statistically characterized using standard deviation across the five repetitions for each batch mass. Preserving all irregularities and fluctuations allowed the model to better capture the incorporation behaviour of the components within the blend and improve its ability to generalize to real-world noisy data. Suppressing these features can lead to the loss of significant information relevant to the mixing process. In addition to instantaneous values and fluctuations, future analyses may also benefit from considering cumulative indicators, such as integrated shear energy (e.g., torque–time integral) and thermal history (e.g., temperature–time integral). These cumulative features can capture the overall mechanical and thermal exposure experienced by the rubber blend during mixing and may provide further insight into the mechanisms influencing the final blend quality. 

### 2.4. GRNN Modelling

The GRNN model was employed to analyse the recorded experimental mixing curves. GRNN is a well-established type of artificial neural network that effectively overcomes several limitations commonly associated with traditional ANNs, such as the requirement for numerous iterations to reach convergence and sensitivity to the initialization of weights and training hyperparameters [10,11,12,13,14,15,16]. Owing to its unique four-layer, feed-forward, memory-based architecture—utilizing Parzen–Rosenblatt probability density estimation with a Gaussian kernel—GRNN enables probabilistic function estimation without iterative training or extensive hyperparameter optimisation. Instead of adjusting weights via backpropagation, the GRNN calculates outputs as weighted averages of target values from training samples, where the weights correspond to the probability density of their distribution [26]. This approach allows rapid and accurate predictions with minimal computational overhead [32]. The GRNN can effectively generalize even from small training datasets and scale well to large volumes of data. It is specifically designed for regression tasks and excels in modelling and predicting complex nonlinear functional relationships between independent and dependent variables [9,26].

Unlike traditional multilayer perceptrons (MLP) or support vector machines (SVM), which rely on iterative weight optimisation using backpropagation and gradient descent, a process that is often computationally intensive and prone to becoming trapped in local minima, GRNNs offer superior stability and reduced sensitivity to weight initialization and convergence issues. Because they are specifically designed to address complex regression problems and are known for their ability to perform well on relatively small datasets, they were considered the most appropriate choice for the current experimental setting. This robustness allows the GRNN to quickly and accurately approximate highly nonlinear functions with complex data structures and variable inputs, which are characteristics typical of mixing processes in the rubber industry. Its ability to rapidly adapt to smaller datasets, generate smooth and stable outputs, and impose minimal computational demands makes it a valuable tool for machine learning, data analysis, modelling, and prediction across a variety of practical and industrial applications [27]. Therefore, it was selected for the intelligent analysis of the real experimental data presented in this study.

The GRNN architecture comprises four interconnected layers that work together to deliver optimal performance [26].

The input layer receives the input data and forwards it to the pattern layer.

The pattern layer uses radial basis functions (RBFs) [12], each centred on a training sample, to quantify the similarity between the current input vector **X** and each training input sample X*i*. This similarity was quantified using the Gaussian kernel of the RBF function [33,34] as follows:

(5) $$\psi_{i}\left(X\right)=exp\left(-\frac{{\left\|X-X_{i}\right\|}^{2}}{2\sigma^{2}}\right),$$

where ${\left\|X-X_{i}\right\|}^{2}\;$ is the squared Euclidean distance *D_i_*^2^ between the current input **X** and the training sample **X***_i_* and *σ* is the smoothing parameter (spread or kernel width) that controls the width of the *ψ*_i_ functions and influences the model’s generalization capability.

The summation layer calculates two sums: a weighted sum and an unweighted sum of the pattern-layer outputs.

The output layer produces the predicted output of the GRNN model as the ratio of these two sums.

(6) $$\hat{Y}\left(X\right)=\frac{\sum_{i=1}^{n}Y_{i}\psi_{i}\left(X\right)}{\sum_{i=1}^{n}\psi_{i}\left(X\right)},$$

where **Y***_i_* is the known target vector for the training sample *i*, *ψ_i_*(**X**) are the weighting functions, and **Ŷ(X)** represents the predicted output vector corresponding to the input **X**.

**Figure 1:**
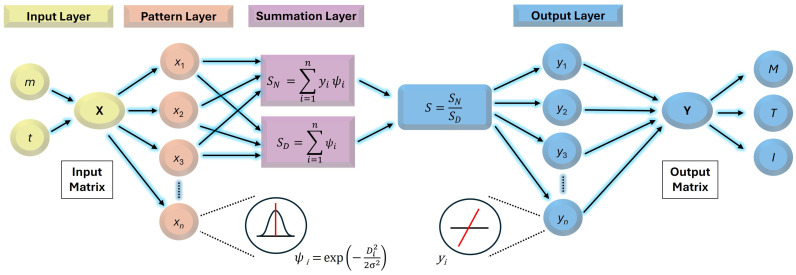
Principal schematic of the GRNN structure.

A principal schematic of the GRNN model structure designed in this study is shown in Figure 1.

### 2.5. GRNN Training

All the experimental mixing curves of the investigated rubber blend were modelled using a well-trained GRNN, which served as a universal function approximator. In this study, the network models the relationships between the torque (**M**), blend temperature (**T**), and electric current (**I**) as dependent variables, with mixing time (**t**) as the independent variable and the mass of the mixed material (**m**) as a parameter of variable dependency across multiple levels. The GRNN-modelled relationship is expressed as follows:

(7) $${\hat{Y}}^{\left(j\right)}={GRNN}_{\sigma }\left(X,Y,\sigma \right),\;\;\;j=1,2,\ldots ,N_{Y},$$

where ${\hat{Y}}^{\left(j\right)}\;$ is the predicted vector for the *j*-th dependent variable (i.e., **M**, **T**, or **I**), *N*_Y_ is the number of dependent variables, and *σ* is the smoothing parameter of the GRNN.

Training the GRNN involves an iterative process aimed at determining the optimal value of the smoothing parameter *σ*, which ensures the best prediction performance. To achieve this, a *k*-fold cross-validation method is employed [35]. This technique splits a dataset into *k* equal-sized parts (folds). In each iteration, the model was trained on *k*−*1* parts and validated on the remaining part. This process is repeated *k* times such that each part serves as the validation set exactly once, ensuring that all data contribute to both training and validation. This enhances the robustness of the model evaluation.

The goal of GRNN training is to optimise *σ* within a predefined positive interval ⟨*σ_min_*, *σ_max_*⟩, constrained to values not exceeding 1. The optimal value σ* minimizes the model prediction error, which is evaluated using the root-mean-squared error (RMSE). The selection of σ* is based on minimizing the average RMSE across all validation folds as follows:

(8) $$\sigma^{*}=arg\;\underset{\sigma_{i}}{\min}\left(\frac{1}{k}\sum_{l=1}^{k}RMSE\left(Y^{\left(j\right)},{\hat{Y}}^{\left(j\right)}\left(X,\sigma_{i}\right)\right)\right).$$

The optimisation interval ⟨*σ_min_*, *σ_max_*⟩ was defined to ensure a balance between accurate prediction and generalization to unseen data. Very small values of σ* can lead to overfitting, where the model captures noise instead of relevant patterns, whereas very large values can result in underfitting, where the model fails to capture the essential structure of the data [26].

## 3. Results and Discussion

### 3.1. Experimental Results

**Figure 2:**
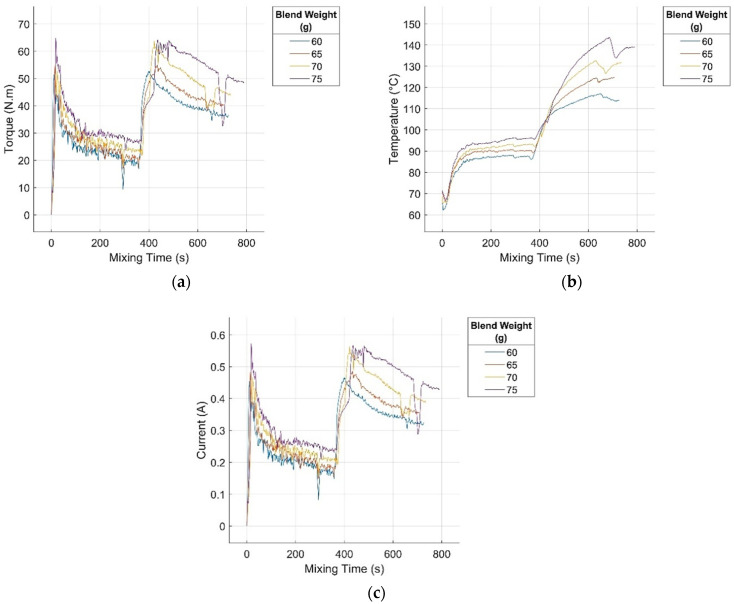
Experimental functional dependencies of (**a**) torque, (**b**) blend temperature, and (**c**) electric current on mixing time for various blend masses in the mixer chamber.

The experimental mixing curves of the investigated rubber blend, depicting the functional dependence of the torque, blend temperature, and electric current on the mixing time for various blend masses in the mixer chamber, are presented in Figure 2a–c.

The torque curves in Figure 2a exhibit a pronounced initial rise, corresponding to the introduction of the rubber matrix into the mixer chamber and initiation of the mixing process upon chamber closure. This nonlinear increase in torque at the early stage of mixing is attributed to inertial effects arising from the mass of the polymer matrix chains, which resist the initial deformation and mechanical motion as they interact with the mixer walls and blades [36]. Both the maximum torque and time required to reach it increased nonlinearly with the blend mass or matrix content in the formulation. Similarly, the initial nonlinear drop in temperature observed in Figure 2b reflects the response of the temperature sensor to the introduction of the cooler ambient-temperature blend matrix into the preheated mixer chamber maintained at a constant mixing temperature (Section 2.2). The minimum temperature and the time required to reach it also increase nonlinearly with the matrix content, which is consistent with the increase in heat capacity and the governing principles of heat transfer [37]. The nonmonotonic trends observed in this stage of mixing may be attributed to the inherent inhomogeneity of natural rubber, which contains varying admixtures and impurities within samples from the same batch, as well as its relatively high polydispersity [38].

Following the peak torque, a nonlinear decrease was observed, primarily owing to a reduction in the viscosity of the blend. This reduction stems from shearing forces, rupture of rubber chains, and disentanglement of macromolecular networks, leading to a decreased resistance to flow. A secondary cause is the concurrent temperature rise (Figure 2b) resulting from frictional heating and deformation of the polymer chains, as well as the accumulation of degradation products [39]. Both the minimum torque and the time required to reach it increased nonlinearly with increasing matrix content. Despite these changes, the minimum time required to adequately mix the matrix before the incorporation of additional blend components, as indicated by the slope of the linear section of the torque curve, remains nearly unaffected by the blend mass. The linear decline in torque indicates a stable degradation rate of the polymer chains, homogeneous dispersion of degradation products within the mixer, and uniform dissipation of mechanical energy. The quasi-stable temperature observed in this phase (Figure 2b) suggests that the linear torque decline is governed primarily by the isothermal rearrangement of the blend, which increases conformational entropy [39].

In the subsequent mixing phase, zinc oxide (ZnO) was added as a sulfur vulcanization activator in accordance with the blend formulation. Given its low concentration (<5 phr, Table 1), this addition had a negligible influence on the torque and temperature curves. This was followed by the addition of stearic acid, which reacts with ZnO to form zinc stearate, a compound that functions as a vulcanization activator. Stearic acid, a wax-like solid with a melting point above 60 °C [40], reduces the blend viscosity when melted, resulting in a sharp torque decrease and the formation of a characteristic V-shaped trough. With increasing blend mass, the magnitude of this viscosity drop diminishes and the time required to reach it increases, both following a monotonic nonlinear trend. After the incorporation of stearic acid, the torque returned to pre-addition values and resumed its original linear decreasing trend. The width of the V-shaped trough, which represents the rate of activator incorporation, remained nearly constant with respect to the blend mass. Owing to the limited sensitivity of the temperature sensor to rapid viscosity changes, the characteristic trough was only slightly visible in the temperature curve (Figure 2b).

In the next mixing phase, carbon black was introduced, resulting in a rapid rise in blend viscosity, torque, and temperature owing to the stiffening effect of the filler on the flow behaviour. Carbon black particles form secondary van der Waals interactions with rubber chains, establishing sliding pseudo-linkages and a secondary polymer network that enhances the resistance to deformation and increases the blend temperature. As these linkages dissipate mixing energy and enhance filler–matrix interactions through improved contact area and adhesion, the filler becomes more uniformly distributed within the matrix [3]. The maximum torque during this phase increases monotonically with the blend mass, whereas the time to reach this maximum shows a non-monotonic trend. The inflection points observed in some torque and temperature curves may reflect temporary reductions in the filler feed rate caused by the accumulation of material near the mixer inlet, which obstructs filler incorporation and distribution.

After reaching the peak, the torque decreased monotonically as the temperature continued to increase, and both trends became linear in the final mixing stage. This suggests that no further significant microstructural transformations occurred, with filler agglomerates fully dispersed and integrated into the matrix. Torque changes are primarily governed by temperature-dependent viscosity reduction. These temperature increases result from continued energy dissipation and structural rearrangement within the matrix–filler system, driving the blend toward maximum conformational entropy [36]. The slope of the linear torque and temperature trends and the time required to reach them display a non-monotonic relationship with increasing blend mass, likely because of the aforementioned rubber inhomogeneity and polydispersity [1].

During the linear torque-reduction phase, sulfur and TBBS were introduced, constituting the sulfur vulcanization system for the rubber matrix. V-shaped troughs were observed consistently across all recorded curves—torque, blend temperature, and electric current—typically between 600 and 750 s, resulting solely from the temporary interruption of the mechanical and thermal conditions caused by opening the mixer chamber to manually add the vulcanization agents. This operation leads to a sudden drop in the internal pressure and localized cooling, producing transient fluctuations in all monitored signals. These signals exhibited a brief decrease before resuming the expected progression. Rapid and adequate incorporation and dispersion must be ensured to avoid premature curing or scorching of the compound [41]. Because of the low concentration of curing agents (Table 1), their addition did not significantly influence the trends of the measured variables.

The electric current versus mixing time curves, in accordance with Equations (3) and (4), differ from the torque curves only by rescaling the dependent variable. Hence, a detailed analysis is unnecessary.

This comprehensive analysis clearly demonstrated that the progression of the observed mixing curves was significantly influenced by the blend mass. In several stages, the trends exhibited highly nonlinear and non-monotonic behaviour, resulting from complex interactions between blend components, mechanical and thermal processes, chemical reactions, and degradation phenomena. These effects are further modulated by dynamically evolving processes and boundary conditions as well as the intrinsic properties of the rubber matrix.

Given the highly complex and nonlinear nature of the mixing process, analytical modelling is exceedingly difficult, imprecise, or even infeasible. A more practical and accurate approach is the use of a well-trained GRNN, which can effectively capture nonlinear behaviours and predict the process dynamics. The selection of suitable input and output variables that are most influential in the dynamics of the mixing processes is essential, as they play a decisive role in determining the final properties of the blend.

### 3.2. GRNN Model Training and Simulation Results

The parameter value of *k* = 10, commonly adopted for training GRNN models via *k*-fold cross-validation on medium-sized datasets, provided an optimal trade-off between overfitting mitigation and retention of sufficiently large training subsets in each fold [35]. This choice ensured robust and accurate estimation of the smoothing parameter *σ*, thereby maximizing model performance across all evaluated datasets (Section 2.5). The optimised GRNN parameter, determined σ* = 1 × 10^−3^, was subsequently used to train the model on normalized data corresponding to the complete torque–temperature–current mixing curves.

The simulation of the trained GRNN model was conducted using normalized input data from both the full-length curves and partial curve segments. The resulting predicted values of the monitored mixing process parameters were evaluated using standard quantitative metrics to assess the accuracy and reliability [33]. For simulations involving partial segments, only the initial 10% of each curve was employed as input, allowing the model to extrapolate the entire mixing process trajectory from the earliest phase. This approach aimed to evaluate the predictive power of the model and its potential for the early-stage assessment of process quality.

The practical significance of this method lies in its ability to fully or selectively compare actual (experimental) and predicted mixing curves in real time. In scenarios where significant deviations in the key process indicators (torque *M*, temperature *T*, or current *I*) from the predicted values are observed, the system can issue an automatic warning. This enables timely corrective intervention to restore the process to its intended course. In an advanced implementation, such interventions can be executed automatically by a process control system. For typical industrial rubber mixers, the most responsive and effective parameter for on-the-fly adjustment is rotor speed, which can be indirectly regulated via electrical current control [5].

**Figure 3:**
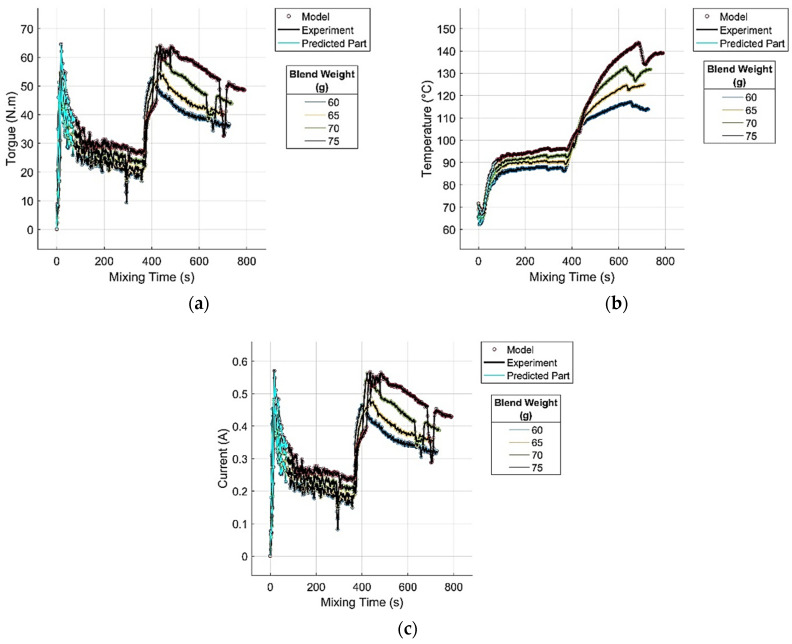
Comparison of experimental mixing curves with GRNN-predicted curves and extrapolated segments for (**a**) torque, (**b**) temperature and (**c**) electric current at varying blend masses.

A comparison between the full experimental mixing curves of the tested rubber blend and their corresponding GRNN predictions, including the extrapolated segments, is presented in Figure 3a–c.

### 3.3. GRNN Model Performance Evaluation and Accuracy Metrics

The quantitative evaluation of the performance of the GRNN model was conducted using two standard statistical metrics, the root mean square error (RMSE) and coefficient of determination (R^2^), defined as follows:

(9) $$RMSE=\sqrt{\frac{1}{n}\sum_{i=1}^{n}{\left(Y_{i}-{\hat{Y}}_{i}\right)}^{2}}$$

and

(10) $$R^{2}=1-\frac{\sum_{i=1}^{n}{\left(Y_{i}-{\hat{Y}}_{i}\right)}^{2}}{\sum_{i=1}^{n}{\left(Y_{i}-\bar{Y_{i}}\right)}^{2}},$$

where $Y_{i}$, ${\hat{Y}}_{i}$ and $\bar{Y_{i}}$ represent the actual values, predicted values generated by the model, and arithmetic mean of the actual values, respectively.

**Figure 4:**
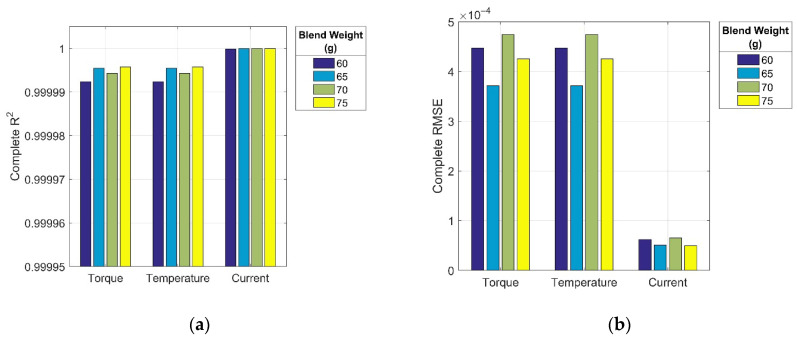
Statistical performance metrics for complete mixing curves: (**a**) coefficient of determination (R^2^), and (**b**) root mean square error (RMSE).

**Figure 5:**
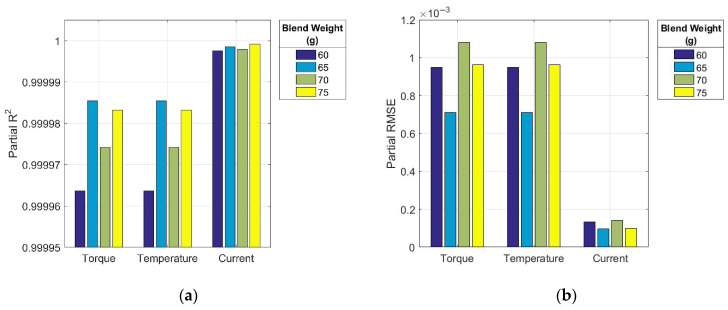
Statistical performance metrics for the initial segments of the mixing curves: (**a**) coefficient of determination (R^2^), and (**b**) root mean square error (RMSE).

The results of the GRNN model’s statistical performance evaluation, expressed through the coefficient of determination (R^2^) and root mean square error (RMSE), are shown in Figure 4a,b for the complete mixing curves and Figure 5a,b for their initial segments.

The analysis of the accuracy metrics revealed that all R^2^ values (Figure 4a and Figure 5a) were very close to or equal to 1. This indicates the excellent ability of the GRNN model to accurately capture the variability of the dependent variables based on independent inputs in all evaluated cases. Although the assessment was performed on the available dataset that was also used for training, the very high R^2^ values reflect the model’s strong learning capacity and suitability for capturing the nonlinear relationships inherent in the mixing process data. Since the optimisation interval ⟨*σ*_min_, *σ*_max_⟩ for the GRNN spread parameter was selected to minimize the risk of both overfitting and underfitting (Section 2.5), the obtained results further support the model’s ability to learn robust patterns with good generalization potential. Nevertheless, its predictive performance for the initial curve segments was slightly lower than that of the complete curves.

The extremely low RMSE values in all cases (Figure 4b and Figure 5b) indicated that the average deviation between the predicted and actual values constituted only a small fraction of the total data range. This confirms the potential of the model for highly accurate and consistent predictions, positioning it as a promising AI-based tool for real-time decision support through intelligent analysis of experimental process data. It should be noted that the substantially lower RMSE values observed for the electric current are attributable to its inherently narrower numerical range compared to the other two monitored process parameters.

### 3.4. GRNN Model Scalability and Adaptability

The training algorithm of the GRNN model, along with the preprocessing of experimental data and their organization into structured datasets (Section 2.3 and Section 2.5), enables flexible adjustment of the number of input and output variables, as well as the number and levels of controllable process parameters. This flexibility makes the model a universal tool for simulating and accurately approximating the progression of experimental curves based on the defined input conditions. As such, it is applicable not only in rubber or polymer blending processing but also in related industrial sectors such as plastics, food, pharmaceutical, chemical, paper, and cosmetic industries—anywhere that precise control over the time-dependent behaviour of technological processes is required.

A key advantage of the GRNN model is its ability to easily adapt the input and output variables, variable parameters, or sensor-based inputs according to the type of material being processed, specific processing conditions, and the given technology, without requiring changes to the core model architecture. It only requires retraining by using real data from the target environment.

The fundamental strength of the GRNN is its universal function approximation capability, which allows it to model complex, nonlinear, and sometimes non-monotonic relationships in data. Once well trained on representative datasets, the model provides optimal or near-optimal predictions. This generalization enables applications beyond rubber blending to fundamentally different technological processes (such as curing of concrete under various climatic conditions) because GRNN learns the input–output mapping directly from data without presupposing underlying physics [42,43].

To maintain the prediction accuracy when applying the GRNN to new materials, processes, or conditions, it is essential to include all relevant process variables and environmental factors as inputs, gather sufficiently diverse and representative data covering the expected operating ranges, properly normalize the input data to ensure meaningful distance metrics, and retrain or adjust the model’s smoothing parameter (*σ*) accordingly.

Although the core GRNN architecture remains unchanged, increasing the input dimensionality or dataset size may lead to higher computational and memory requirements. These can be mitigated through data reduction or clustering techniques, balancing GRNN’s flexibility with the practical implementation constraints.

Although the model is theoretically scalable, this study did not empirically test its performance on larger datasets, higher parameter dimensionality, or more complex input spaces. Similarly, while retraining is expected to be straightforward, no simulation of model adaptation to alternative blend formulations or different thermal regimes has been carried out. These are promising avenues for future research.

Thus, the proposed intelligent process control and monitoring system based on the GRNN model represents an efficient, scalable, and easily implementable solution with low computational demands and strong potential for real-world industrial deployment.

## 4. Conclusions

This study developed and experimentally validated an intelligent predictive system to support real-time decision making in the control of rubber blend mixing processes. The system is based on a GRNN model, optimised using 10-fold cross-validation, and trained to accurately predict the evolution of key process parameters—torque (as a proxy for viscosity), temperature, and electric current—across varying blend masses based on natural rubber.

The GRNN model demonstrated outstanding predictive performance not only for the full mixing curves but also when simulating the process using only the initial 10% of the measured data. This early-stage prediction capability enables timely diagnostics and dynamic optimisation of the mixing process in real time. The model achieved extremely low RMSE and R^2^ values that were consistently close to 1, confirming its precision, robustness, and capacity to reconstruct and extrapolate time-dependent experimental trends based on partial inputs. Although the prediction was performed using data from the same experimental records used for training, the model effectively demonstrated its ability to generalize from limited input, which is essential from a practical perspective.

The proposed system is suitable for industrial deployment as a robust and scalable solution to intelligent quality control, productivity enhancement, and real-time monitoring. Owing to its inherent flexibility, the model can be easily adapted to broader applications, requiring rapid and reliable analysis of experimental process data and autonomous data-driven decision support.

Importantly, each monitored parameter contributes uniquely to process optimisation; torque reflects the mechanical behaviour and viscosity changes during mixing, temperature provides critical information about thermal conditions to prevent premature curing, and electric current indicates the mechanical load and mixing dynamics, enabling the detection of consistency changes in the blend. Integrating these dependencies allows for comprehensive and effective control of the mixing parameters, thereby improving process stability and product quality.

It is also important to emphasize that the proposed methodology represents a significant advancement over the existing literature by enabling real-time predictive optimisation based on partial input data. Unlike conventional static models, this system dynamically links the evolution of the process parameters to actionable adjustments, making it particularly suitable for modern data-driven manufacturing environments.

Nevertheless, the current study did not empirically evaluate the adaptability of the model to fundamentally different material compositions, process types, or environmental conditions, such as alternative blend formulations or varied thermal regimes. Future research should address these aspects to confirm the generalizability and scalability of this approach.

Finally, the accuracy and practical utility of the model critically depend on the availability of comprehensive and representative experimental datasets covering the expected operating ranges. When sufficiently diverse data are available, the GRNN-based system has strong potential to reduce production variability and improve process efficiency across a wide range of industrial sectors.

## Figures and Tables

**Table 1:** Rubber blend formulation.

Materials	Contents (phr)	Function	Producer
Natural rubber grade 1500	100	Matrix	Synthos Kralupy a.s., Kralupy nad Vltavou, Czech Republic
Carbon black type N550 (CB)	35	Filler	Makrochem Sp. z o.o., Lublin, Poland
Zinc oxide (ZnO)	3	Vulcanization activator	SlovZink a.s., Koseca, Slovakia
Stearic acid	1	Vulcanization activator	Setuza a.s., Ústí nad Labem, Czech Republic
Sulfur Crystex OT33 (S)	1.75	Vulcanizing agent	Eastman Chemical company, Kingsport, TN, USA
TBBS	1	Vulcanization accelerator	Duslo a.s., Šaľa, Slovakia

## Data Availability

The original contributions presented in this study are included in the article. Further inquiries can be directed to the corresponding author.
